# Effect of cluster set resistance training combined with high-intensity interval training on cardiorespiratory and muscular fitness in untrained young men

**DOI:** 10.7717/peerj.20492

**Published:** 2025-12-19

**Authors:** Jing Ma, Rongze Ye, Shumin Bo, Yang Cheng

**Affiliations:** Capital University of Physical Education and Sports, Haidian, Beijing, China

**Keywords:** Cluster set resistance training, High-intensity interval training, Untrained young men, Physical fitness

## Abstract

**Objective:**

This study aims to investigate the impact of cluster set resistance training combined with high-intensity interval training (CSRT-HIIT) on the body composition, cardiovascular and muscular fitness of untrained young men.

**Methods:**

Twenty-two participants were randomly assigned to the CSRT-HIIT or traditional resistance training combined with high-intensity interval training (TRT-HIIT). Both groups had cardiorespiratory and muscular fitness measured before and after the intervention.

**Results:**

Compared to the baseline, significant increases were observed in the 1RM weights for squat, bench press, deadlift, and rowing, as well as the maximum training volume at 70% 1RM, maximum oxygen uptake, standing long jump, and the thickness of the pectoralis major, biceps brachii, and rectus femoris in both the CSRT-HIIT and TRT-HIIT groups after the intervention, with a significant decrease in body fat percentage (*P* < 0.05). No statistically significant differences were found between the groups for these variables (*P* > 0.05).

**Conclusion:**

Twelve weeks of both CSRT-HIIT and TRT-HIIT significantly improved cardiorespiratory and muscular fitness in untrained young men, demonstrating that the novel CSRT-HIIT is an equally effective alternative to traditional TRT-HIIT.

## Introduction

With the rapid development of China’s economy and society and the acceleration of urbanization, the physical activity patterns of young people have undergone structural changes, with sedentary behavior becoming increasingly prevalent. A cross-national study covering 23 countries found that approximately 40% of university students fail to meet the World Health Organization’s recommended levels of physical activity (PA). This phenomenon is particularly pronounced in developing and middle-income countries, such as China, where the proportion of university students with insufficient physical activity reaches as high as 51.5% (Pengpid et al., 2015). Insufficient physical activity poses a dual threat to both cardiorespiratory function and musculoskeletal health (Bu et al., 2021). Cardiorespiratory fitness (CRF) is widely recognized as the gold standard for assessing cardiovascular health, with substantial clinical value. Extensive research has demonstrated that low levels of CRF are not only an independent risk factor for cardiovascular disease and coronary mortality (Kodama et al., 2009), but have also been elevated by the American Heart Association (AHA) to the status of a “fifth vital sign,” underscoring its significant association with all-cause mortality (Ross et al., 2016). Concurrently, the health of the skeletal muscular system is equally critical. The ratio of skeletal muscle mass to body weight has been shown to correlate strongly with survival rates in major diseases (Liu et al., 2025), and it functions as a dynamic endocrine organ that regulates communication between organs and controls the nervous, metabolic, and skeletal systems (Zunner et al., 2022). In response, physical activity has been confirmed as an effective non-pharmacological intervention capable of simultaneously enhancing cardiorespiratory fitness and preserving skeletal muscle health, thereby reducing health risks in sedentary populations (Mi et al., 2025). Therefore, exploring exercise interventions that can synergistically improve both cardiorespiratory and musculoskeletal health is of great scientific significance and practical value for promoting the overall health of physically inactive young adults.

The World Health Organization recommends that adults perform at least 150–300 min of moderate-intensity aerobic exercise, or 75–150 min of high-intensity aerobic exercise, or an equivalent combination of moderate and high-intensity aerobic exercise, per week. In addition, muscle strength training should be performed at least 2 days per week (Tracey & Holmes, 2021). For individuals with no exercise experience, traditional resistance training combined with high-intensity interval training (TRT-HIIT) has been demonstrated to be an effective method for improving physical fitness (Li et al., 2024a). However, the continuous intra-set movement pattern characteristic of traditional resistance training (TRT) within TRT-HIIT protocols can induce significant acute fatigue, leading to considerable physiological and psychological stress, which may pose a substantial challenge for beginners or those returning to exercise (Li et al., 2024a).

Cluster set resistance training (CSRT), as a novel form of resistance training, incorporates rest periods both between sets and within sets (Tufano, Brown & Haff, 2017). This contrasts with TRT, which only provides rest after each set while performing exercises continuously within the set, potentially leading to fatigue accumulation and a decline in exercise quality as repetitions increase. Jukic et al. (2022) found that compared with TRT, CSRT can effectively alleviate the fatigue experienced by men who have undergone resistance training and enhance their power output, thereby ensuring the quality of training. Additionally, Mao et al. (2023) demonstrated that an 8-week moderate-intensity (70–75%) CSRT protocol was more effective in improving lower limb muscle strength and power in young male subjects than TRT.

The above research indicates that CSRT, which utilizes intra-set intermittent recovery, effectively alleviates acute physical and mental fatigue as well as subjective stress. Furthermore, this approach helps maintain power output stability, ensures movement quality, and reduces the risk of exercise-related injuries. For beginners in particular, this more accessible training model not only optimizes the single-session exercise experience but also significantly enhances long-term adherence by minimizing perceived exertion and discomfort (Mao et al., 2023). Based on the aforementioned evidence, we hypothesize that a protocol combining CSRT with high-intensity interval training (CSRT-HIIT) may enhance the body’s adaptation to high-intensity stimuli by promoting intra-session recovery. This approach is expected to not only improve overall training benefits but also offer a safer exercise option for untrained individuals. However, the actual training effects of CSRT-HIIT remain unclear.

Therefore, this study aims to investigate the effects of a 12-week CSRT-HIIT intervention on muscle function and cardiorespiratory fitness in untrained young males, comparing it with TRT-HIIT, in order to provide a theoretical basis and practical reference for exercise program selection in this population.

## Materials and Methods

### Subjects

Twenty-six untrained young men meeting the experimental requirements were recruited from a university in Beijing. During the experimental intervention, four participants withdrew for personal reasons, leaving a total of 22 participants for the final analysis. Subjects’ inclusion criteria were: aged between 18 and 28 years; no smoking and drinking habits; no regular physical activity within the past year, and a Physical Activity Rating Scale (PARS-3) ≤ 19 (Eklund et al., 2016; Yang et al., 2021). Exclusion criteria were: presence of fracture or joint strain and sprain within 6 months; suffering from contraindications to exercise such as asthma, heart disease, and tumors; and participation in experiments by other researchers.

All participants voluntarily signed an informed consent form. The ethics committee of Capital University of Physical Education and Sports approved this study (Approval number: 2024A027). An independent samples T-test revealed no significant differences in the basic information between the two groups before the intervention. The basic information of the subjects before the intervention is tabulated in Table 1.

**Table 1:** Anthropometric data for participants.

Parameter	TRT-HIIT (*n* = 11)	CSRT-HIIT (*n* = 11)	*P*
Age (years)	21.5 ± 2.98	22.15 ± 2.7	0.10
Height (cm)	174.1 ± 6.5	178.7 ± 6.6	0.46
Weight (kg)	72.9 ± 7.0	76.7 ± 4.35	0.23
Total caloric intake (kcal)	1,802 ± 586	1,905 ± 694	0.724
Carbohydrate (g)	231.4 ± 86.2	225.2 ± 61.5	0.852
Protein (g)	81.5 ± 33.8	84.3 ± 45.9	0.973
Fat (g)	38.8 ± 10.9	39.0 ± 12.1	0.968

**Note:**

TRT-HIIT, Traditional resistance training combined with high-intensity interval training; CSRT-HIIT, cluster set resistance training combined with high-intensity interval training.

### Exercise protocol

**Figure 1:**
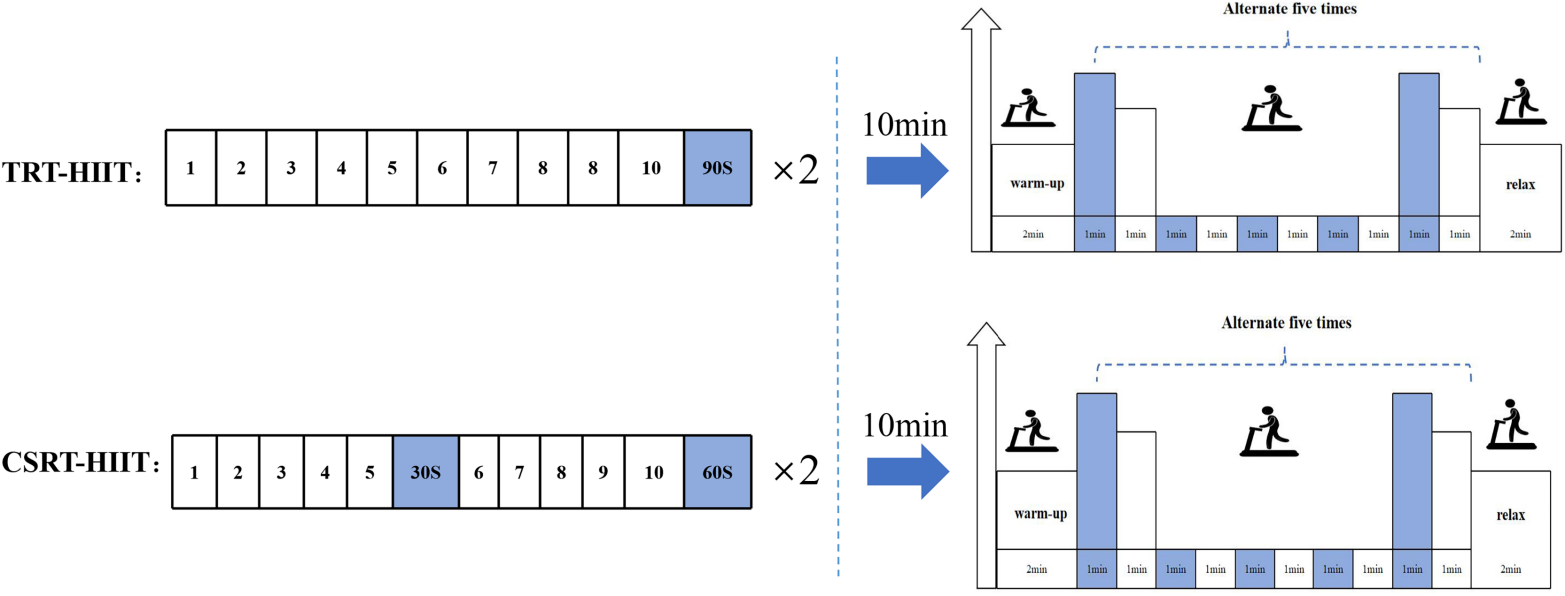
Schematic diagram of the TRT-HIIT and CSRT-HIIT intervention protocols. The sequence and structure of the two combined exercise interventions. TRT-HIIT Protocol: The resistance training component consists of traditional resistance training (TRT), including four exercises (squats, bench presses, deadlifts, and rows). Each exercise is performed for 2 sets of 10 repetitions at 70% of 1-repetition maximum (1RM), with a 90-s rest interval between sets and a 2–3 min rest interval between exercises. The subsequent HIIT component includes a 3-min warm-up, followed by five cycles of 1-min high-intensity intervals (at 85–95% VO_2_max) alternating with 1-min low-intensity recovery (at 30–35% VO_2_max), and concludes with a 2-min cool-down. CSRT-HIIT Protocol: The resistance training component employs cluster set resistance training (CSRT), using the same four exercises, total sets, and repetitions as TRT. The key difference is the intra-set rest: each set of 10 repetitions is divided into two clusters of five repetitions, separated by a 30-s brief rest interval. The rest interval between sets is 60 s, and between exercises is 2–3 min. The HIIT component is identical in structure and intensity to the one performed in the TRT-HIIT protocol.

To ensure participant safety and efficacy during the subsequent training intervention, and to minimize potential measurement errors stemming from initial discomfort, all study participants underwent a 1-week adaptation phase. This period was designed to familiarize participants with the training protocol, ensure proper execution of fundamental movement patterns, and acclimatize them to initial physical demands. Following confirmation of stable participant status post-adaptation, baseline data collection was performed. Subsequently, a 12-week intervention training program was initiated. The required sample size was determined *a priori* using G*Power software (version 3.1.9.7). The analysis was based on parameters from similar studies (Karsten et al., 2021; Mao et al., 2023), including an alpha level of 0.05, a target statistical power (1 − β) of 0.80, and an effect size of 0.4. This calculation indicated a minimum requirement of 16 participants (8 per group). To account for potential attrition and enhance the study’s robustness, we initially enrolled 26 participants. Following the collection of baseline measurements, participants were randomly allocated in a 1:1 ratio to either the CSRT-HIIT group (*n* = 13) or the TRT-HIIT group (*n* = 13) using a computer-generated randomization sequence. This was achieved by assigning participants a unique number from 1 to 26; those numbered 1–13 were assigned to the CSRT-HIIT group, and those numbered 14–26 were assigned to the TRT-HIIT group. During the intervention period, four participants withdrew for personal reasons. Consequently, the final analysis was conducted on a total of 22 participants, with 11 in each group. The intervention protocol for the TRT-HIIT group referred to a previous report (Li et al., 2024a). TRT consists of four exercises: squats, bench presses, deadlifts, and rows, performed at 70% of 1RM for 2 sets, 10 repetitions per set, and a 90-s rest interval between sets. The rest interval between exercises is 2–3 min. After 10 min of TRT intervention, HIIT is conducted. The HIIT intervention protocol is as follows: a 3-min warm-up before starting the exercise, followed by 1 min of high-intensity running at 85–95% VO_2_max, then 1 min of low-intensity running at 30–35% VO_2_max, alternating for 5 cycles, and a 2-min cool-down after the exercise (Fig. 1).

The CSRT-HIIT group’s cluster set resistance training protocol is based on a literature report (Merrigan et al., 2020), while the HIIT exercise protocol is the same as that in the TRT-HIIT group. The four exercises, number of sets, and repetitions per set in CSRT are the same as TRT, but with a 30-s brief rest interval inserted within each set after the 5th repetition, followed by completing the remaining 5 repetitions, then a 60-s rest interval between sets, and a 2–3 min rest interval between exercises. After 10 min of CSRT intervention, HIIT is conducted (Fig. 1).

### Measurement

#### Anthropometric

Height and weight were measured with an ultrasonic height and weight meter. Body composition was analyzed using an InBody body composition analyzer, measuring parameters such as skeletal muscle mass, percent body fat, and lean body mass.

#### Regional muscle thickness

Professional medical imaging personnel used the LOGIQ Book XP portable ultrasound device to test the muscle thickness of the pectoralis major, biceps brachii, and rectus femoris in the participants. The measurements were carried out by the same professional medical imaging technician, and this technician was unaware of the subject’s group assignment information. Before measurement, use a soft ruler to accurately position and mark the measurement area: the measurement area for the biceps brachii is from the acromion process of the scapula to the distal 60% of the lateral epicondyle of the humerus; the measurement area for the pectoralis major is between the third and fourth ribs below the midpoint of the clavicle; the measurement area for the rectus femoris is at the midpoint between the anterior superior iliac spine and the lower edge of the patella. After applying the ultrasonic-specific coupling agent to the measurement area, place the ultrasonic probe vertically onto the measurement site, but do not press on the skin. To reduce muscle congestion caused by exercise, participants were required to cease vigorous exercise for at least 72 h before image collection. Three images were collected for each muscle group, and the two most similar images were selected for averaging during analysis. All measurements were completed by the same professional medical imaging technician and were evaluated using Intraclass Correlation Coefficients (ICC) (Mao et al., 2023).

#### Maximum strength

The maximum strength of the upper and lower limbs was assessed using the one-repetition maximum test for flat bench press, deadlift, bent-over row, and back squat. The tests followed the guidelines published by the NSCA and were supervised, monitored, and supported by coaches certified by the NSCA. Before the test, participants performed a 5-min slow jog, followed by dynamic stretching and muscle activation exercises. During the warm-up phase, subjects performed 8 to 10 repetitions using 50% of the estimated 1RM, followed by two sets of 2 to 3 repetitions using 60% to 80% of the estimated 1RM. Subsequently, the weight was gradually increased until the subject could complete no more than one repetition to determine the 1RM. The rest period between sets was 3 to 5 min.

#### Muscular endurance

The muscular endurance of the upper and lower body was assessed through the maximum number of repetitions at 70% of 1RM in the squat, bench press, deadlift, and rowing exercises, before and after the intervention. Participants were required to perform the exercises with proper form and as much concentric movement speed as possible until muscle fatigue, defined as when the weight stopped moving or the participant failed to maintain proper technique. Any repetitions without a full range of motion were not counted.

#### Standing long jump

The standing long jump is used to determine the explosive strength of the lower limbs. The tester will accurately measure the vertical distance from the jumping line to the nearest point of landing, if any part of the body other than the feet touches the ground, it will be regarded as an invalid test. Each person jumps 3 times, taking the highest score as the final result.

#### Maximum oxygen uptake

VO_2_max was measured using the Bruce Incremental Load Exercise Test protocol (Bruce, Kusumi & Hosmer, 1973). Participants should not engage in strenuous exercise for 24 h before the test, and the test should be performed at least one hour after a meal. Participants will be required to sign an informed consent form before the start of the experiment. To monitor fatigue, the rate of perceived exertion (RPE, 6–20 Borg’s scale) was assessed after each stage and at the end of the test, while heart rate (HR) was recorded simultaneously using a coded transmitter belt (Polar H10, Finland) (Liu et al., 2022). VO_2_max will be determined based on any three of the following four conditions: (1) the heart rate reaches the predicted maximum or no longer rises with increasing intensity of exercise; (2) the respiratory quotient meets or exceeds 1.15; (3) a plateau or decline in the oxygen uptake curve; and (4) a subjective fatigue score of no less than 18.

#### Diet control

During the experiment, participants were asked to maintain their original diet and exercise habits. One week before and after the intervention, participants were required to record their diet for 24 h on three consecutive days to calculate their total caloric intake as well as protein, fat, and carbohydrate intake *via* the Mint Health app (Shanghai, China) (Benítez-Flores et al., 2019; Ugurlu et al., 2024).

### Statistical analyses

The experimental data were organized using Microsoft Excel software, and continuous variables were described by means and standard deviations. Data were processed and analyzed by SPSS 27.0 software (IBM Corp., Armonk, NY, USA) and an independent samples t-test was used to assess differences between groups at baseline. If there were between-group differences in the baseline data, the baseline data were used as covariates, and analysis of covariance was performed to test for differences. The Shapiro-Wilk test was used to assess whether the data conformed to a normal distribution. If the data conformed to a normal distribution, two-way ANOVA (group × time) was employed to analyze within-group and between-group changes as well as interaction effects. In cases where significant interaction effects were observed, Bonferroni *post hoc* tests were conducted for further analysis. If the interaction effect was not significant, the within-group changes were analyzed by a paired-samples t-test. If the data did not conform to normal distribution, the Wilcoxon signed rank sum test and the Kruskal-Wallis rank sum test were used to compare within- and between-group differences.

## Results

### Body composition and regional muscle thickness

For body weight, skeletal muscle mass (Table 2), the group-by-time interaction, the main effect of group, and the main effect of time were not significant between the CSRT-HIIT and TRT-HIIT groups (*P* > 0.05).

**Table 2:** Test indicators and their change rates in young men before and after intervention.

	TRT-HIIT		CSRT-HIIT		ANOVA (P)		
	Pre	Post	Pre	Post	G	T	G × T
Body weight (kg)	74.60 ± 7.90	72.90 ± 7.53	78.64 ± 5.80	75.99 ± 6.93	0.182	0.386	0.710
Skeletal muscle (kg)	33.86 ± 5.12	33.93 ± 4.90	37.59 ± 3.00	37.15 ± 4.11	0.111	0.841	0.788
Body fat percentage (%)	18.93 ± 3.97	17.15 ± 4.82*	17.75 ± 4.61	14.68 ± 5.03*	0.379	0.004	0.420
Biceps brachii (cm)	1.60 ± 0.22	1.99 ± 0.41**	1.47 ± 0.39	1.95 ± 0.53**	0.581	<0.001	0.637
Pectoralis major (cm)	1.47 ± 0.38	1.95 ± 0.53**	1.37 ± 0.15	1.93 ± 0.32**	0.713	<0.001	0.639
Rectus femoris (cm)	1.90 ± 0.15	2.31 ± 0.17**	2.01 ± 0.29	2.43 ± 0.14**	0.113	<0.001	0.934
VO2max (ml/kg/min)	45.9 ± 6.22	52.78 ± 5.21**	45.56 ± 4.38	52.52 ± 3.77**	0.897	<0.001	0.933
Squat (kg)	113.18 ± 34.52	154.32 ± 29.37**	111.36 ± 21.57	158.64 ± 21.34**	0.912	<0.001	0.322
Bench Press (kg)	64.55 ± 23.07	80.68 ± 21.62**	64.55 ± 17.81	81.36 ± 13.98**	0.923	<0.001	0.936
Deadlift (kg)	119.18 ± 29.88	145.00 ± 27.02**	111.55 ± 22.43	148.00 ± 24.67**	0.830	<0.001	0.090
Rowing (kg)	68.27 ± 15.6	89.91 ± 11.83**	69.00 ± 12.69	96.09 ± 11.84**	0.510	<0.001	0.221
Standing Long Jump (cm)	233.73 ± 25.20	245.36 ± 25.09**	252.19 ± 26.95	264.72 ± 23.64**	0.089	<0.001	0.827
Squat Training Volume (kg)	910.64 ± 323.77	1,753.82 ± 617.44**	984.77 ± 386.59	1,610.32 ± 398.68**	0.631	<0.001	0.799
Bench Press Training Volume (kg)	553.00 ± 304.64	655.30 ± 270.70**	595.95 ± 238.49	715.91 ± 225.94*	0.799	0.004	0.631
Deadlift Training Volume (kg)	870.74 ± 370.06	1,311.86 ± 525.17*	964.73 ± 267.38	1,183.32 ± 303.09*	0.901	0.001	0.211
Rowing Training Volume (kg)	596.21 ± 269.72	991.77 ± 286.04**	668.25 ± 290.42	871.95 ± 180.78**	0.822	<0.001	0.066

**Notes:**

Compared with before intervention.

* *P* < 0.05.

** *P* < 0.01.

For body fat percentage (Table 2), the time-by-group interaction effect (F = 0.826, *P* = 0.379, *η^2^p* = 0.056) and the main effect of group (F = 0.689, *P* = 0.420, *η^2^p* = 0.047) were not significant between the CSRT-HIIT and TRT-HIIT groups, however, the main effect of time was significant (F = 11.501, *P* = 0.004, *η^2^p* = 0.451). After the intervention, body fat percentage significantly decreased in both the TRT-HIIT group (*P* = 0.050,) and the CSRT-HIIT group (*P* = 0.039).

For biceps brachii muscle thickness (Table 2), the time-by-group interaction effect (F = 0.320, *P* = 0.581, *η*^*2*^*p* = 0.022) and the main effect of group (F = 0.232, *P* = 0.637, *η*^*2*^*p* = 0.016) were not significant between the CSRT-HIIT and TRT-HIIT groups, however, the main effect of time was significant (F = 32.658, *P* < 0.001, *η*^*2*^*p* = 0.700). After the intervention, the biceps muscle thickness significantly increased in both the TRT-HIIT group (*P* = 0.003) and the CSRT-HIIT group (*P* = 0.007).

For pectoralis major muscle thickness (Table 2), the time-by-group interaction effect (F = 0.230, *P* = 0.639, *η*^*2*^*p* = 0.016) and the main effect of group (F = 0.230, *P* = 0.639, *η*^*2*^*p* = 0.016) were not significant between the CSRT-HIIT and TRT-HIIT groups, however, the main effect of time was significant (F = 39.833, *P* < 0.001, *η*^*2*^*p* = 0.740). After the intervention, the pectoralis major muscle thickness significantly increased in both the TRT-HIIT group (*P* = 0.007) and the CSRT-HIIT group (*P* = 0.001).

For rectus femoris muscle thickness (Table 2), the time-by-group interaction effect (F = 0.007, *P* = 0.934, *η*^*2*^*p* = 0.001) and the main effect of group (F = 2.863, *P* = 0.113, *η*^*2*^*p* = 0.170) were not significant between the CSRT-HIIT and TRT-HIIT groups, however, the main effect of time was significant (F = 38.047, *P* < 0.001, *η*^*2*^*p* = 0.731). After the intervention, the rectus femoris muscle thickness significantly increased in both the TRT-HIIT group (*P* = 0.004) and the CSRT-HIIT group (*P* = 0.003).

### Maximum oxygen uptake

For VO_2_max (Table 2), the time-by-group interaction effect (F = 0.007, *P* = 0.933, *η*^*2*^*p* = 0.000) and the main effect of group (F = 0.017, *P* = 0.897, *η*^*2*^*p* = 0.001) were not significant between the CSRT-HIIT and TRT-HIIT groups, however, the main effect of time was significant (F = 47.177, *P* < 0.001, *η*^*2*^*p* = 0.713). After the intervention, VO_2_max significantly increased in both the TRT-HIIT group (*P* = 0.004) and the CSRT-HIIT group (*P* < 0.001).

### Muscle fitness

#### Muscle maximal strength

For squat maximum strength (Table 2), the time-by-group interaction effect (F = 1.033, *P* = 0.332, *η*^*2*^*p* = 0.049) and the main effect of group (F = 0.012, *P* = 0.912, *η*^*2*^*p* = 0.001) were not significant between the CSRT-HIIT and TRT-HIIT groups, however, the main effect of time was significant (F = 214.396, *P* < 0.001, *η*^*2*^*p* = 0.915). After the intervention, the squat maximum strength of both the CSRT-HIIT group (*P* < 0.001) and the TRT-HIIT group (*P* < 0.001) increased significantly.

For bench press maximum strength (Table 2), the time-by-group interaction effect (F = 0.007, *P* = 0.936, *η*^*2*^*p* = 0.001) and the main effect of group (F = 0.009, *P* = 0.923, *η*^*2*^*p* = 0.001) were not significant between the CSRT-HIIT and TRT-HIIT groups, however, the main effect of time was significant (F = 131.662, *P* < 0.001, *η*^*2*^*p* = 0.868). After the intervention, the squat maximum strength of both the CSRT-HIIT group (*P* < 0.001) and the TRT-HIIT group (*P* < 0.001) increased significantly.

For deadlift maximum strength (Table 2), the time-by-group interaction effect (F = 3.181, *P* = 0.090, *η*^*2*^*p* = 0.137) and the main effect of group (F = 0.047, *P* = 0.830, *η*^*2*^*p* = 0.002) were not significant between the CSRT-HIIT and TRT-HIIT groups, however, the main effect of time was significant (F = 109.031, *P* < 0.001, *η*^*2*^*p* = 0.845). After the intervention, the deadlift maximum strength of both the CSRT-HIIT group (*P* < 0.001) and the TRT-HIIT group (*P* < 0.001) increased significantly.

For rowing maximum strength (Table 2), the time-by-group interaction effect (F = 1.593, *P* = 0.221, *η*^*2*^*p* = 0.074) and the main effect of group (F = 0.451, *P* = 0.510, *η*^*2*^*p* = 0.022) were not significant between the CSRT-HIIT and TRT-HIIT groups, however, the main effect of time was significant (F = 127.159, *P* < 0.001, *η*^*2*^*p* = 0.864). After the intervention, the deadlift maximum strength of both the CSRT-HIIT group (*P* < 0.001) and the TRT-HIIT group (*P* < 0.001) increased significantly.

#### Muscle explosive force

For standing broad jump (Table 2), the time-by-group interaction effect (F = 0.049, *P* = 0.827, *η^2^p* = 0.002) and the main effect of group (F = 3.202, *P* = 0.089, *η^2^p* = 0.138) were not significant between the CSRT-HIIT and TRT-HIIT groups, however, the main effect of time was significant (F = 34.671, *P* < 0.001, *η^2^p* = 0.634). After the intervention, the standing long jump significantly increased in both the CSRT-HIIT group (*P* < 0.001) and the TRT-HIIT group (*P* = 0.004).

#### Muscle endurance

For squat endurance (Table 2), the time-by-group interaction effect (F = 0.961, *P* = 0.339, *η^2^p* = 0.046) and the main effect of group (F = 0.051, *P* = 0.824, *η^2^p* = 0.003) were not significant between the CSRT-HIIT and TRT-HIIT groups, however, the main effect of time was significant (F = 43.776, *P* < 0.001, *η^2^p* = 0.686). After the intervention, the squat maximum strength of both the CSRT-HIIT group (*P* < 0.001) and the TRT-HIIT group (*P* = 0.008) increased significantly.

For bench press endurance (Table 2), the time-by-group interaction effect (F = 0.067, *P* = 0.799, *η*^*2*^*p* = 0.003) and the main effect of group (F = 0.238, *P* = 0.631, *η*^*2*^*p* = 0.012) were not significant between the CSRT-HIIT and TRT-HIIT groups, however, the main effect of time was significant (F = 10.582, *P* = 0.004, *η*^*2*^*p* = 0.346). After the intervention, bench press endurance significantly increased in both the TRT-HIIT group (*P* = 0.003) and the CSRT-HIIT group (*P* = 0.047).

For deadlift endurance (Table 2), the time-by-group interaction effect (F = 1.668, *P* = 0.211, *η*^*2*^*p* = 0.077) and the main effect of group (F = 0.016, *P* = 0.901, *η*^*2*^*p* = 0.001) were not significant between the CSRT-HIIT and TRT-HIIT groups, however, the main effect of time was significant (F = 14.658, *P* = 0.001, *η*^*2*^*p* = 0.423). After the intervention, bench press endurance significantly increased in both the TRT-HIIT group (*P* = 0.018) and the CSRT-HIIT group (*P* = 0.012).

For rowing endurance (Table 2), the time-by-group interaction effect (F = 6.864, *P* = 0.066, *η*^*2*^*p* = 0.256) and the main effect of group (F = 0.052, *P* = 0.822, *η*^*2*^*p* = 0.003) were not significant between the CSRT-HIIT and TRT-HIIT groups, however, the main effect of time was significant (F = 66.962, *P < 0.001*, *η*^*2*^*p* = 0.770). After the intervention, bench press endurance significantly increased in both the TRT-HIIT group (*P* < 0.001) and the CSRT-HIIT group (*P* = 0.004).

## Discussion

The findings indicate that 12 weeks of both CSRT-HIIT and TRT-HIIT significantly improved cardiorespiratory fitness and muscular endurance in untrained young men, with no significant differences in overall efficacy between the two protocols. This result failed to support our initial hypothesis that CSRT-HIIT would be superior. Our hypothesis was predicated on the notion that by reducing intra-set density, CSRT would attenuate acute fatigue accumulation, potentially enhancing the quality and cumulative stimulus of the subsequent HIIT component (Haff et al., 2003). Nevertheless, the present study establishes CSRT-HIIT as a viable alternative to TRT-HIIT, given its comparable training adaptations. Furthermore, CSRT is known to induce lower acute fatigue, thereby mitigating physiological and psychological strain, which is particularly beneficial for novices or individuals returning to training (Mao et al., 2023). This lower-fatigue characteristic is conducive to long-term adherence, positioning CSRT-HIIT as an effective and practical training model for untrained young men.

Body composition is a comprehensive indicator that assesses the proportion of various tissues such as fat and muscle in the human body. Aerobic exercise can enhance the body’s oxidative capacity and improve the efficiency of fat energy supply; resistance exercise can stimulate muscle growth and increase lean body mass. The combined application of aerobic and resistance exercises can achieve effective optimization of body composition by synergistically increasing the basal metabolic rate (Bouamra et al., 2022). In our research, after 12 weeks of CSRT-HIIT and TRT-HIIT interventions, there were no significant changes in body weight, skeletal muscle mass and a significant reduction in body fat percentage in young men. This result is similar to the findings of Trowell et al. (2022) that a 10-week combined aerobic and resistance exercise intervention significantly reduced body fat percentage but did not change total body weight and lean body mass in distance runners.

Skeletal muscle hypertrophy is the result of a sustained positive net protein balance, in which the rate of muscle protein synthesis exceeds that of muscle protein breakdown. Research on the mechanisms of exercise-induced muscle growth has primarily focused on two areas: the influence of exercise on changes in circulating hormone levels and the promotion of protein synthesis through cellular signal transduction pathways (Qaisar, Bhaskaran & Van Remmen, 2016). The findings of the present study indicate that 12 weeks of CSRT-HIIT and TRT-HIIT are equally effective in promoting muscle hypertrophy in untrained young men. The underlying mechanism for this equivalence may stem from the inherently similar hypertrophic potential of CSRT and TRT themselves. Zaras et al. (2022) conducted a study on young men with training experience and found that in 7-week high-load (85% 1RM) bench press training, CSRT (4 sets × 6 repetitions, 20-s rest between sets, 3-min rest between groups) and TRT (4 sets × 6 repetitions, 3-min rest between groups) had similar effects on the increase in triceps muscle thickness. However, that study did not analyze the phased adaptive characteristics during the training process, nor did it track long-term outcomes. Therefore, future research is needed to further clarify the differences between these two training modalities in terms of their dynamic responses and long-term adaptations.

Cardiorespiratory fitness involves the efficiency of the heart’s pumping ability, pulmonary gas exchange, oxygen transport capacity, and muscle oxidative metabolism. It is an important indicator for measuring the ability of the cardiovascular and respiratory systems to supply oxygen to muscles during aerobic exercise, and it also reflects the functional efficiency of mitochondria (Lee et al., 2010). VO_2_max is a commonly used indicator for evaluating cardiorespiratory function and the muscle’s ability to utilize oxygen. It is often considered an important parameter for assessing an individual’s aerobic endurance and overall health status (Kaminsky et al., 2017). A study has shown that a 12-week, twice-weekly combination of resistance and aerobic exercise was effective in enhancing VO_2_max and 20 m shuttle run (lap) exercise performance in adolescents (Li et al., 2024b). A similar study also found that a combination of resistance and aerobic exercise 3 times per week for 9 weeks significantly increased VO_2_max and 6-min walk test distance in sedentary, healthy, and obese youths (Bouamra et al., 2022). Consistent with the above results, the 12-week CSRT-HIIT and TRT-HIIT in our study also significantly improved the VO_2_max of young men.

Muscle fitness refers to the body’s ability to achieve resistance to or overcome external forces through the contraction of muscle fibers to maintain and perform dynamic body activities, which is usually reflected in three aspects: muscular strength, muscular endurance, and muscular explosive power. Muscle fitness is not only an indicator of muscle health, but also affects cognitive functions, especially executive ability and learning efficiency (Bao et al., 2024; Isaac, Lima-Filho & Lourenco, 2021). Therefore, muscle health is crucial for the physiological well-being and work/study performance of young men.

Maximum muscular strength is defined as the highest force output an individual can produce during a maximal voluntary contraction. Previous research has demonstrated that a 9-week combined resistance and aerobic training program, performed three times weekly, significantly enhances lower-body maximum strength in moderately active men, irrespective of training sequence (Lee et al., 2020). Similarly, the present study observed that a 12-week intervention with either CSRT-HIIT or TRT-HIIT led to significant increases in maximum muscular strength in young men. The fundamental distinction between these two training protocols lies in their intra-set structure. In TRT, continuous moderate-load sets lead to a gradual decline in the concentrations of high-energy phosphates, such as adenosine triphosphate (ATP) and phosphocreatine (PCr), thereby attenuating maximal force production. In contrast, the short intra-set rest periods (15–30 s) incorporated into CSRT facilitate partial recovery of these energy substrates, which in turn enhances overall training power output and the effectiveness of individual repetitions (Asadi & Ramírez-Campillo, 2016). Furthermore, the intra-set rest strategy in CSRT has been shown to attenuate mechanical fatigue, ratings of perceived exertion, and metabolic stress both during and after training sessions (Jukic et al., 2021). Based on these physiological advantages, we initially hypothesized that CSRT-HIIT would exhibit greater potential than TRT-HIIT for improving the one-repetition maximum (1RM) performance in the squat, bench press, deadlift, and row. However, the findings of this study were not entirely consistent with this hypothesis. We propose that this discrepancy may be attributable to two primary factors. First, the inclusion of HIIT as a potent adaptive stimulus in both protocols may have masked the subtle effects arising from differences in intra-set structure, resulting in the failure to detect statistically significant differences in overall functional capacity improvements between the groups. Second, the training volume in the present intervention was relatively low, which may have been insufficient to fully elicit the potential advantages of CSRT-HIIT for strength development.

Muscle explosive force is the ability of the neuromuscular system to maximize impulse production in a limited period of time and is mainly influenced by muscle fiber type, nervous system excitability, and neural modulation of muscle fibers. Muscle explosiveness is greater when there is a higher proportion of fast muscle fibers, elevated nervous system activity, and effective mobilization of more fast muscle fibers for activity (Häkkinen et al., 2001). Research has shown that an 8-week, 3-times-a-week combination of resistance and aerobic exercise is effective in enhancing explosive force in recreationally trained young men (Ruiz-Alias et al., 2022). The present study found that 12 weeks of both CSRT-HIIT and TRT-HIIT significantly improved muscle explosive strength in young men, consistent with these results. Since there was no significant difference in the improvement of basic muscle strength between the two training programs, this might further lead to similar improvement effects of explosive power induced by the two programs.

Enhanced muscular endurance is associated with multiple physiological mechanisms within skeletal muscle, such as increased glycogen storage, improved oxygen transport efficiency, enhanced muscle buffering capacity, and a metabolic shift toward fatty acid oxidation (Morishima et al., 2020). Previous research has demonstrated that an 8-week combined resistance and aerobic exercise program performed three times per week comprehensively improves muscular endurance in untrained young males (Li et al., 2024a). In the present study, 12 weeks of both CSRT-HIIT and TRT-HIIT improved muscular endurance in young men, consistent with these results. Multiple comparative studies on TRT and CSRT have found that TRT is superior to CSRT in improving muscular endurance (Mao et al., 2023; Jukic et al., 2021). Nevertheless, no significant differences in physiological responses to long-term muscular endurance were observed between TRT-HIIT and CSRT-HIIT in this study. This may be attributed to the equal total rest time between the two protocols, the fact that the CSRT protocol did not require each set to be performed to muscular failure, and the potent adaptive stimulus of HIIT itself, which may have minimized the differences between the two set-structure protocols.

This study has several limitations. First, the sample size calculation was predicated on a relatively large effect size reported in prior research. While this assumption is supported by the literature, it may have constrained our statistical power to detect smaller, yet potentially meaningful, effects. This limitation, combined with the 12-week intervention duration, may have been insufficient to elicit or detect significant differences in cardiorespiratory and muscular fitness between the groups, despite the theoretical physiological advantages of the CSRT-HIIT cluster structure. Second, the absence of surface electromyography to monitor neuromuscular activation and muscle biopsies to analyze fiber-type transitions precluded mechanistic insights into long-term neuromuscular adaptations. Finally, body composition was assessed using bioelectrical impedance analysis (BIA), a method that may offer lower accuracy for body fat and visceral adipose tissue quantification compared to the gold-standard dual-energy X-ray absorptiometry (Wang et al., 2018). This choice may have limited the precision of our findings regarding changes in adiposity. To address these limitations and build upon our findings, future research should prioritize multi-center collaborations to achieve larger sample sizes, implement longer-term intervention protocols, and incorporate comprehensive mechanistic assessments, such as sEMG and muscle biopsies. Such an approach would be essential to validate the potential differential efficacy of CSRT-HIIT and enhance the mechanistic explanatory power of the evidence.

## Conclusions

In conclusion, our findings demonstrate that 12 weeks of both CSRT-HIIT and TRT-HIIT are effective for enhancing cardiorespiratory and muscular fitness in untrained young men. Critically, we observed no significant differences in the overall outcomes between the two training modalities. This indicates that the novel CSRT-HIIT strategy offers benefits comparable to the well-established TRT-HIIT approach. Therefore, CSRT-HIIT represents a viable and scientifically-grounded alternative that can be incorporated into physical training programs to promote overall fitness. Future research should investigate the long-term effects of CSRT-HIIT and its efficacy in different populations.

## Supplemental Information

10.7717/peerj.20492/supp-1 Supplemental Information 1 Raw data on cardiorespiratory fitness and muscle health indices in untrained young men before and after a 12-week intervention combining clustered resistance training with high-intensity interval training.

## Additional Information and Declarations

### Data Availability

The following information was supplied regarding data availability:

The raw measurements are available in the Supplemental File.
